# Using an Ishikawa diagram as a tool to assist memory and retrieval of relevant medical cases from the medical literature

**DOI:** 10.1186/1752-1947-5-120

**Published:** 2011-03-29

**Authors:** Kam Cheong Wong

**Affiliations:** 1 University of Sydney, Sydney Medical School, NSW, Australia; 2 University of Western Sydney, School of Medicine, NSW, Australia; 3 Beyond Medical Education, NSW/VIC, Australia; 4 George Street Medical Practice, Bathurst, NSW, Australia

## Abstract

Studying medical cases is an effective way to enhance clinical reasoning skills and reinforce clinical knowledge. An Ishikawa diagram, also known as a cause-and-effect diagram or fishbone diagram, is often used in quality management in manufacturing industries.

In this report, an Ishikawa diagram is used to demonstrate how to relate potential causes of a major presenting problem in a clinical setting. This tool can be used by teams in problem-based learning or in self-directed learning settings.

An Ishikawa diagram annotated with references to relevant medical cases and literature can be continually updated and can assist memory and retrieval of relevant medical cases and literature. It could also be used to cultivate a lifelong learning habit in medical professionals.

## Introduction

Doctors are accustomed to learning from their more experienced peers as well as from their own experiences in treating their patients [1]. Because of this, it is important that they develop learning techniques that are proactive and encourage a lifelong learning orientation. Case reports can provide valuable sources of information for others to learn from. Studying medical cases is an effective way to enhance clinical reasoning skills and reinforce clinical knowledge [2]. A case report provides important and detailed information about a patient that is often lost in larger studies [3]. Reading case reports is also intellectually stimulating. When clinicians or medical students analyze a clinical problem, they usually start with potential common causes. For example, if a patient presents with secondary amenorrhea, a clinician will consider common causes such as pregnancy and use of contraceptive medications before exploring other less common but critical causes such as hyperprolactinemia, ovarian cancer and so on.

When clinicians are faced with a puzzling clinical problem, they may search journals that publish clinical cases for information about the condition [4]. There are various sources for medical cases such as the *Journal of Medical Case Reports*, *BMJ Case Reports *and the *New England Journal of Medicine*. However, because of the diversity of the case reports, it may be difficult to recall and organize the located material in a systematic manner in order to explain a clinical problem. Ishikawa diagrams are an efficient way of organizing case reports in a clinical setting.

## Methods

The Ishikawa diagram was invented by Kaoru Ishikawa, who pioneered quality management techniques in Japan in the 1960 s. The diagram is considered one of the seven basic tools of quality control [5]. It is also known as a fishbone diagram because of its shape. The 'fish head' represents the main problem. The potential causes of the problem, usually derived from brainstorming sessions or research, are indicated in the 'fish bones' of the diagram.

**Figure 1:**
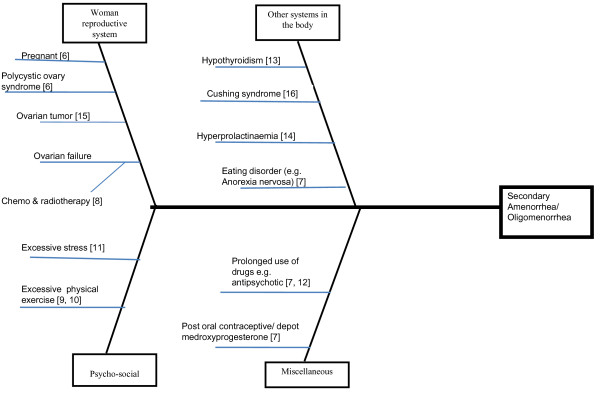
**Ishikawa diagram**. 'Woman's reproductive system' includes causes such as pregnancy, polycystic ovary syndrome, ovarian tumor, and ovarian failure. 'Other systems in the body' includes causes such as hypothyroidism, Cushing syndrome, hyperprolactinemia, and eating disorders. 'Psychosocial' includes causes such as excessive stress and excessive physical exercise. 'Miscellaneous' includes causes such as prolonged use of drugs, for example anti-psychotics, and after oral contraceptive/depot medroxyprogesterone treatment.

As an example for illustration, 'secondary amenorrhea/oligomenorrhea' has been chosen as the main presenting problem. 'Secondary amenorrhea/oligomenorrhea' is indicated in the head of the Ishikawa diagram (Figure 1). When searching for the potential causes of the main presenting problem, one can either work in a team with others or in a self-directed learning setting. Clinicians would conduct brainstorming sessions and search the relevant journals to find potential causes for secondary amenorrhea/oligomenorrhea, listing them on a whiteboard or flipchart. The list would then be reviewed to extract relevant causes in the context of the main presenting problem. These causes would then be organized in the 'fish bones' of an Ishikawa diagram (Figure 1). There is no limit to the number of 'fish bones' in the diagram. Each 'fish bone' can be subdivided into smaller 'bones' if necessary to show the relationship of all potential causes to the presenting problem. For example, 'chemotherapy and radiotherapy' are indicated in the branch of the 'fishbone' that shows the cause of ovarian failure, a potential cause for secondary amenorrhea/oligomenorrhea (Figure 1). The cited references for the relevant case reports and literatures are also indicated in the Ishikawa diagram so that readers can retrieve the case reports and relevant literatures easily.

The potential causes for secondary amenorrhea/oligomenorrhea have been identified and categorized in four groups related to 'women's reproductive systems', 'other systems in the body', 'psychosocial', and 'miscellaneous, for example drugs'. The causes include pregnancy [6], polycystic ovarian syndrome [6], amenorrhea after oral contraceptive/depot medroxyprogesterone treatment [7], eating disorder (for example, anorexia nervosa) [7], premature ovarian failure [8], excessive physical exercise [9,10], excessive stress [11], prolonged use of anti-psychotic [7,12], hypothyroidism [13], hyperprolactinemia [14], ovarian cancer [15], and Cushing syndrome [16]. Thus the Ishikawa diagram illustrates and summarizes the potential causes for secondary amenorrhea/oligomenorrhea (Figure 1).

## Conclusions

Rare but critical cases should be studied and included in an Ishikawa diagram to remind clinicians of relevant information during their clinical reasoning processes. For example, the *Journal of Medical Case Reports *has published the case of a 22-year-old lactating woman who presented with four months of amenorrhea associated with signs of virilization. The patient was diagnosed as having an androgen secreting steroid cell tumor of the ovary [15]. In addition, *BMJ Case Reports *has published a case demonstrating the relationship between hypothyroidism and secondary amenorrhea. Important learning points are highlighted: serum thyroid stimulating hormone (TSH) should be measured in every adolescent with menstrual irregularities, multicystic ovaries as a presenting manifestation of juvenile hypothyroidism is a rare occurrence and represents advanced disease, and appropriate diagnosis and levothyroxine replacement therapy is effective and it can prevent inadvertent surgery [13].

Furthermore, the reader should appraise the published case to assess the credibility of the information and should look for updated information in the future. For example, if the readers are not fully convinced of the explanation for the pathophysiology of 'specificity spill over' phenomenon that may contribute to multicystic ovaries [13,17], he or she should search for more information about it and look out for future publications on this topic. Information gathered from other sources can be included in the diagram as well, such as the paper published in the *British Journal of Obstetrics and Gynaecology*, which has substantiated information about ovarian cancers and amenorrhea [8]. In this way, continually organizing and updating information on an Ishikawa diagram can cultivate lifelong learning habits in medical professionals.

Medical educators can also apply Ishikawa diagrams to facilitate problem-based learning when teaching medical students and junior doctors. Starting with a clinical vignette, facilitators can help medical students and junior doctors to identify the main presenting problem of a patient, conduct brainstorming sessions and search in the literature to find the potential causes, then categorize these causes in an Ishikawa diagram. The Ishikawa diagram can then be kept by individual learners for continual updating when they acquire new or relevant information. In short, an Ishikawa diagram can assist memory and the retrieval of relevant medical case reports and literatures.

## Competing interests

The author declares that he has no competing interests.

## References

[B1] Jenicek M Clinical case reporting in evidence-based medicine 2001 2 London: Arnold

[B2] Vandenbroucke JP In defense of case reports and case series Ann Intern Med 2001 134 330 334 11182844 10.7326/0003-4819-134-4-200102200-00017

[B3] Kidd M Hubbard C Introducing journal of medical case reports J Med Case Reports 2007 1 1 10.1186/1752-1947-1-1 17411446 PMC1839763

[B4] Wong G Case reports: a helping hand to generalists J Med Case Reports 2008 2 311 10.1186/1752-1947-2-311 18822140 PMC2561039

[B5] Ishikawa K Loftus JH (Eds) Introduction to quality control 1990 Tokyo, Japan: 3A Corporation

[B6] Golden NH Carlson JL The pathophysiology of amenorrhea in the adolescent Ann N Y Acad Sci 2008 1135 163 178 10.1196/annals.1429.014 18574222

[B7] Gordon CM Functional hypothalamic amenorrhea New Engl J Med 2010 363 365 371 10.1056/NEJMcp0912024 20660404

[B8] Schmidt KT Larsen EC Andersen CY Andersen AN Risk of ovarian failure and fertility preserving methods in girls and adolescents with a malignant disease BJOG 2010 117 163 174 10.1111/j.1471-0528.2009.02408.x 19874293

[B9] Meczekalski B Podfigurna-Stopa A Warenik-Szymankiewicz A Genazzani AR Functional hypothalamic amenorrhea: current view on neuroendocrine aberrations Gynecol Endocrinol 2008 24 4 11 10.1080/09513590701807381 18224538

[B10] Nattiv A Loucks AB Manore MM Sanborn CF Sundgot-Borgen J Warren MP American College of Sports Medicine position stand. The female athlete triad Med Sci Sports Exerc 2007 39 1867 1882 10.1249/mss.0b013e318149f111 17909417

[B11] Liu JH Hypothalamic amenorrhea: clinical perspectives, pathophysiology, and management Am J Obstet Gynecol 1990 163 1732 1736 2122729 10.1016/0002-9378(90)91437-h

[B12] Perkins RB Hall JE Martin KA Neuroendocrine abnormalities in hypothalamic amenorrhea: spectrum, stability, and response to neurotransmitter modulation J Clin Endocrinol Metab 1999 84 1905 1911 10.1210/jc.84.6.1905 10372685

[B13] Bhansali A Shanmugasundar G Walia R Santosh R Dutta P Acute abdomen and hypothyroidism BMJ Case Reports 2009 10.1136/bcr.12.2008.1356 PMC3029595 21686354

[B14] Srikantha M Butterworth R Pharmacological hyperprolactinaemia BMJ Case Reports 2009 10.1136/bcr.01.2009.1432 PMC3030289 22140413

[B15] Haji AG Sharma S Babu M Vijaykumar D Chitrathara K Androgen secreting steroid cell tumor of the ovary in a young lactating women with acute onset of severe hyperandrogenism: a case report and review of literature J Med Case Reports 2007 1 182 10.1186/1752-1947-1-182 18088412 PMC2231374

[B16] Lado-Abeal J Rodriguez-Arnao J Newell-Price JD Perry LA Grossman AB Besser GM Trainer PJ Menstrual abnormalities in women with cushing's disease are correlated with hypercortisolemia rather than raised circulating androgen levels J Clin Endocrinol Metab 1998 83 3083 3088 10.1210/jc.83.9.3083 9745407

[B17] Yoshimura M Hershman J Thyrotropic action of human chorionic gonadotropic Thyroid 1995 5 425 434 10.1089/thy.1995.5.425 8563483

